# Introduction to “Chronic Rhinosinusitis and Concomitant Medical Disorders”

**DOI:** 10.3390/medsci8010017

**Published:** 2020-03-23

**Authors:** David A. Gudis

**Affiliations:** Chief, Division of Rhinology & Anterior Skull Base Surgery; Department of Otolaryngology–Head and Neck Surgery, Columbia University Irving Medical Center, New York, NY 10032, USA; dag62@cumc.columbia.edu

It is with great pleasure and enthusiasm that we present to you this Special Issue of Medical Sciences. In this issue, we present a comprehensive and contemporary review of the relationship between chronic rhinosinusitis and other respiratory disorders and, moreover, how our medical and surgical interventions as otolaryngologists impact those respiratory conditions. Our understanding of chronic rhinosinusitis has evolved tremendously over the last two decades. As we have learned, chronic rhinosinusitis—a chronic inflammatory condition of the nasal cavity and paranasal sinuses—is often a local inflammatory response to a systemic or mucosal disorder. The underlying systemic medical conditions not only influence the presentation and diagnosis of chronic rhinosinusitis, but also modify patients’ responses to medical and surgical interventions. Chronic rhinosinusitis associated with cystic fibrosis, for example, is a disorder quite distinct from that associated with aspirin-exacerbated respiratory disease. 

A clear understanding of the nuances that distinguish these unique and challenging disorders is critical for the practicing otolaryngologist. Equally important, however, is a clear understanding of the powerful benefits that our interventions as otolaryngologists can have for our patients’ rhinologic and systemic health. Knowing that our rhinologic interventions might spare an asthma patient a trip to an emergency room, or reduce lung infections in a cystic fibrosis patient, makes this a very exciting time to be a rhinologist. 

This compendium includes basic science, translational, and clinical evidence demonstrating the pathophysiology and treatment outcomes of inflammatory respiratory disorders. Dr. Kanda and colleagues examine the impact of eosinophilic inflammation, seen in clinical disorders such as asthma and aspirin-exacerbated respiratory disease, on airway epithelium in a murine model [1]. Dr. Safi et al. [2] and Dr. Zheng et al. [3] respectively explore the contemporary medical and surgical management paradigms of cystic fibrosis, in addition to treatment impact on pulmonary and systemic health. Dr. Massoth and colleagues [4] discuss the relationship between asthma and chronic rhinosinusitis, and Dr. Li and colleagues [5] further examine aspirin-exacerbated respiratory disease. Finally, Dr. Belcher et al. [6] and Dr. Marcus et al. [7] explore the common comorbidities of adenoiditis and allergic rhinitis, respectively, and how such concomitant disorders may impact the presentation, management, and outcomes of these patients.

We hope you enjoy this Special Issue of Medical Sciences.
